# Performance evaluation of presepsin using a Sysmex HISCL‐5000 analyzer and determination of reference interval

**DOI:** 10.1002/jcla.24618

**Published:** 2022-07-23

**Authors:** Taewon Kang, Jeaeun Yoo, Hyunyu Choi, Seungok Lee, Dong Wook Jekarl, Yonggoo Kim

**Affiliations:** ^1^ Departement of Laboratory Medicine, Seoul St. Mary's Hospital, College of Medicine The Catholic University of Korea Seoul South Korea; ^2^ Research and Development Institute for In Vitro Diagnostic Medical Devices of Catholic University of Korea, College of Medicine The Catholic University of Korea Seoul South Korea; ^3^ Departement of Laboratory Medicine, Incheon St. Mary's Hospital, College of Medicine The Catholic University of Korea Seoul South Korea

**Keywords:** analytical evaluation, biomarker, diagnosis, presepsin, sepsis

## Abstract

**Background:**

Analytical evaluation of newly developed presepsin by a Sysmex HISCL‐5000 (Sysmex, Japan) automated immune analyzer was performed.

**Methods:**

For evaluation, sepsis patient samples were collected before treatment in an emergency department. Precision, linearity, limit of blank/limit of detection, method comparisons, and reference intervals were evaluated. Method comparisons were performed using a PATHFAST immune analyzer (LSI Medience Corporation, Japan).

**Results:**

Precision using a 20x2x2 protocol for low (306 pg/mL) and high (1031 pg/mL) levels resulted in within‐laboratory standard deviation (95% confidence interval [CI]) and coefficient of variation (CV) %, which were as follows: 15.3 (13.1–18.7), 5.5% and 47.7, (40.5–58.1), 6.4%, respectively. Linearity using patient samples and calibrators were measured from 201 to 16,177 and 188 to 30,000 pg/mL, respectively. The regression equation was y = −23.2 + 1.008x (SE = 162.4) for low levels and y = 779.9 + 1.006x (SE = 668) for high levels. Method comparison by Passing–Bablock analysis was as follows: y = −209.77 + 1.047x (S_yx_ = 335.3). The correlation coefficient (95% CI) was 0.869 (0.772–0.927) with statistical significance (*p* < 0.001). Reference intervals from 120 normal healthy subjects showed that 300 pg/mL was the cut off. Presepsin tended to show a higher value at higher ages and in males. Presepsin showed correlation with some parameters, and the correlation coefficient (*p* value) were as follows: hematocrit, 0.198 (0.03); eGFR (CKD‐EPI), −0.240 (0.0129); MDRD‐eGFR, −0.194 (0.048), respectively.

**Conclusion:**

Presepsin measurement by HISCL‐5000 showed reliable performance. Further clinical studies are required for the diagnosis and prognosis of sepsis.

## Graphical abstract

Reference interval is required to interpret biomarkers for clinical decision making. Presepsin values was influcenced by various factors. Presepsin values from healthy subjects showed positive correlation with age. Presepsin values was also positively correlated with hematocrit, but negatively correlated with calculated eGFR (CKD‐EPI) and MDRD eGFR. Male showed higher values compared to females.

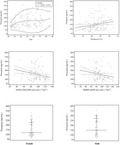

## 1 INTRODUCTION

Sepsis is defined as organ dysfunction caused by an infectious agent along with host immune dysregulation.^1^ Revision of the sepsis definition narrowed the boundary of sepsis compared to the former definition by requiring organ dysfunction based on a sequential organ failure assessment (SOFA) scoring system.^2^ As the diagnosis of sepsis requires laboratory and clinical data, a quick SOFA (qSOFA) scoring system could be applied outside the intensive care unit and considers mental status, a respiratory rate equal to or greater than 22/minute, and systolic blood pressure less than or equal to 100 mm/Hg. Although qSOFA could be useful for initial screenings, the clinical course of sepsis is fast, and diagnosis of sepsis based on the definition is complicated.

Biomarkers that reflect systemic responses during sepsis have been studied continuously, and there are various surrogate markers for the diagnosis of sepsis.^3^, ^4^, ^5^, ^6^ Procalcitonin (PCT), C‐reactive protein (CRP), cytokines, and chemokines have been studied extensively under the previous and revised sepsis definitions. Among them, a soluble CD14 molecule (presepsin) has been developed and applied toward the diagnosis and prognosis of sepsis.^7^, ^8^, ^9^, ^10^, ^11^

Presepsin is a soluble CD14 molecule that is cleaved and released into the general circulation after activation by a monocyte or macrophage.^7^ Pathogen‐associated molecular species such as lipopolysaccharide (LPS) from gram‐negative bacteria can bind to serum lipoprotein‐binding protein, both of which bind to CD14. These molecules can bind to toll‐like receptors to initiate intracellular signaling. Cleavage of CD14 after LPS binding can produce various CD14 fragments including presepsin. As presepsin was produced during the innate immunity process, this molecule was studied for diagnosis and prognosis of sepsis and showed varied results compared to those of PCT.^7^, ^8^

Recently, an automated immunoassay using a chemiluminescent method was developed to measure presepsin. In this study, the analytical performance of presepsin was studied along with a reference interval and compared with other automated methods.

## 2 MATERIALS AND METHODS

### 2.1 Patient

This study was approved by the Institutional Review Board of Seoul and Incheon St. Mary's Hospital, The Catholic University of Korea, Seoul, Korea. Samples from sepsis patients visiting the emergency department were collected before a treatment. Sepsis was diagnosed using a SOFA score with suspected bacterial infection.^1^, ^2^

Presepsin was measured using the chemiluminescence enzyme immunoassay method by HISCL‐5000 immune analyzer (Sysmex, Kobe, Japan).^12^, ^13^ Biotin was labeled on the anti‐presepsin antibody, which captures presepsin. That complex attaches to streptavidin‐coated magnetic bead which binds alkaline phosphatase labeled antibody. ALP reacts with luminescent substrate (CDP‐star) for photon emission.^13^ Turn‐around‐time for measuring the first sample was 17 min and 10–30 s for the following samples. The total processing capacity was around 200 tests per hour.

### 2.2 Presepsin evaluation

#### 2.2.1 Precision

Precision was evaluated according to CLSI guideline EP05‐A3 for duplicated samples, two times a day, for 20 working days.^14^ Low level (306 pg/mL) and high level (1031 pg/mL) quality control (QC) material provided by the manufacturer were evaluated. These QC materials (Lot QNPS‐012) were reconstituted according to the manufacturer's insert and used accordingly. Target value (range) of Level 1 (QNPS‐112) and Level 2 (QNPS‐212) was as follows: 315 pg/mL (252–378); 1034 pg/mL (827–1241), respectively. Repeatability, between‐run, between day, and within‐ laboratory coefficient of variation (CV) was analyzed.^15^

#### 2.2.2 Linearity

Linearity was analyzed according to CLSI guideline EP06‐A.^16^ Seven serum concentrations were prepared from patients as mixtures of low (201 pg/mL) and high (16,177 pg/mL) levels as follows: 0.165 high (H) + 0.835 low (L); 0.350H + 0.650 L; 0.5H + 0.5 L; 0.650H + 0.350 L, 0.835H + 0.165 L. In addition, as high values from patient samples were rare for linearity evaluation, calibration materials assigned as 188 and 30,000 pg/mL from manufacturer were measured for linearity validation using seven concentration of serum. First‐, second‐, and third‐order polynomial regression analysis was performed. The allowable non‐linearity for deviation from linearity was defined as an intra‐individual biological variation of 22.3%.^17^

#### 2.2.3 LOB/LOD

Limit of blank (LOB) / limit of detection (LOD) values were measured according to CLSI guideline EP‐17‐A2.^18^ The LOB was measured using four blank sample pools (C1–C4) and two levels of concentration sample pools (C5–C6) for presepsin. All samples were measured 20 times over three working days. C5–C6 were sample diluents for LOD measurements claimed by manufacturers. The LOB was calculated as follows: LOB ≤ mean of blank samples (μ of C1–C4) minus 1.64 * standard deviation of the blank (σ of C1–C4). The LOD was calculated as follows: LOD = LOB +1.645 σs (σs, standard deviation of lowest concentration).

#### 2.2.4 Method comparison

Method comparison was performed according to CLSI guideline EP09‐A2.^19^ The sample volumes of 10–30 uL are required for presepsin. For method comparison, 87 samples from suspected sepsis patients were collected within 24 hours from blood drawn and refrigerated at −80°C for method comparison in duplicate. During measurement, three samples were clotted and discarded before measurements. A total of 84 plasma from EDTA samples was analyzed by both Sysmex HISCL‐5000 and PATHFAST (LSI Medience Corporation,), which is a compact chemiluminescent immunoassay system. For Sysmex HISCL‐5000, the measurement range was from 0 to 30,000 pg/mL and for PATHFAST, the measurement range was from 0 to 20,000 pg/mL. For method comparison, samples from 0 to 20,000 pg/mL were selected and a Passing–Bablock regression analysis was performed to obtain a linear fit. Bland–Altman analysis was performed and plotted to analyze the agreement between the two methods.

#### 2.2.5 Reference interval

Reference intervals were analyzed according to CLSI C28‐A3.^20^ For the determination of reference interval, a direct approach was performed which was based on a posteriori selection of samples from routine health checkup subjects.^21^ Subjects without underlying disease or prescribed medication from electronic medical records were selected and that resulted in 124 subjects.

Age distribution were as follows: 21–30 (*n* = 19); 31–40 (*n* = 17); 41–50 (*n* = 25); 51–60 (*n* = 25); 61–70 (*n* = 24); 71 and above (*n* = 14), respectively. Four samples were regarded as outliers by the Tukey method and were omitted from further analysis. Laboratory data from 120 subjects were used. Hematological parameters were measured using a Sysmex XN2000 (Sysmex), and parameters related to blood chemistry were measured using a Roche Cobas c702 (Roche Diagnostics, Basel, Swtizerland).

Two estimated glomerular filtration rates (eGFR) were calculated. Chronic kidney disease epidemiology collaboration eGFR and eGFR (CKD‐EPI) values were calculated as follows: 141 * min (serum creatinine/kappa, 1)^α^ * max (serum creatinine/kappa, 1)^‐1.209^ *0.993^age^ * 1.018 (if female) * 1.159 (if black). Kappa was 0.7 in females and 0.9 in males. Alpha was −0.329 in females and − 0.411 in males.^22^, ^23^ Isotope dilution mass spectrometry (IDMS) traceable eGFR using modification of diet in renal disease (MDRD‐eGFR) were calculated using the following equation: MDRD‐eGFR = 175 * serum creatinine^‐1.154^ * age^‐0.263^ * (0.742 if female) * (1.212 if black).^22^, ^24^

As the measured values showed a non‐normal distribution by the Shapiro–Wilk test (W = 0.8856, *p* < 0.001), non‐parametric robust methods using percentiles were used to calculate reference and confidence intervals using bootstrapping with 10,000 repeats.^25^

#### 2.2.6 Clinical presepsin data

To support that the renal function was related to higher presepsin concentration, presepsin data were collected during 2020–2021 from the hospital electrical medical record and sorted by the department. We discarded the departments with less than 10 patients and the pediatric department. We hypothesized that the nephrology division might show higher presepsin concentrations.

#### 2.2.7 Statistical analysis

R software version 3.4.4 (R Foundation for Statistical Computing, Vienna, Austria) was used for precision calculations, linearity analysis, and LOB/LOD calculations. MedCalc for Windows, version 18.2.1 (MedCalc Software, Mariakerke, Belgium) was used for method comparisons and reference interval calculations.

## 3 RESULTS

### 3.1 Precision

The precision of low‐level material for within‐run, between‐run, between‐day, and within‐laboratory SD (95% CI) and CV values are listed in Table 1. The CV values of within‐laboratory and total precisions were 5.5% and 6.4% for low and high presepsin levels, respectively.

**TABLE 1:** Precision of presepsin. Low and high presepsin concentrations were evaluated as duplicates, twice a day for 20 working days (20x2x2 protocol)

	Low level	High level
Mean (pg/mL)	306	1031
Repeatability		
SD (95% CI)	6.7 (5.5–10.5)	17.8 (14.6–27.8)
CV (%)	2.2	5.8
Between‐run		
SD (95% CI)	2.5 (2.08–3.25)	26.7 (21.9–34.2)
CV (%)	0.8	8.7
Between‐day		
SD (95% CI)	13.5 (11.1–17.4)	35.3 (29.1–45.2)
CV (%)	4.4	11.5
Within‐laboratory		
SD (95% CI)	15.3 (13.1–18.7)	47.7 (40.5–58.1)
CV (%)	5.5	6.4

Abbreviation: CI, confidence interval; SD, standard deviation; CV, coefficient of variation.

### 3.2 Linearity

**FIGURE 1:**
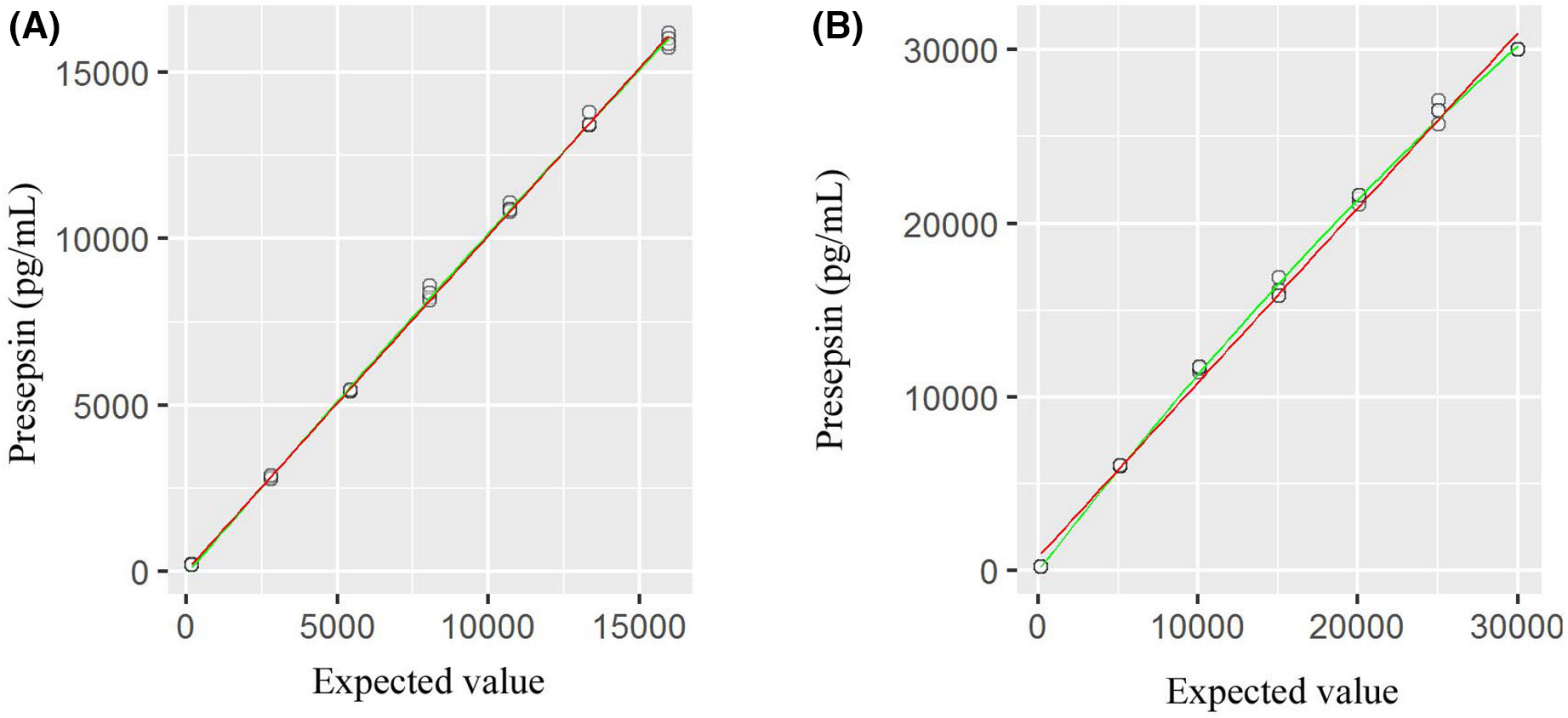
Linearity of presepsin using patient samples (A) and a calibrator (B). The regression line was y = 23.2 + 1.008x (R^2^ = 0.9991, SE = 162.4) (A) and y = 779.9 + 1.006x (R^2^ = 0.9959, SE = 668) (B), respectively. The red and green line denotes regression lines with linear and nonlinear fit, respectively

As the claimed linear range was wide, we prepared seven diluted samples from patients and a calibrator. Linearity was measured from 201 to 16,177 pg/mL for patient samples (Figure 1). The regression equation was y = 23.2 + 1.008x (*R* ^2^ = 0.9991, standard error [SE] = 162.4). Additional linearity was measured using a calibrator from 188 to 30,000 pg/mL. The regression equation was y = 779.9 + 1.006x (*R* ^2^ = 0.9959, SE = 668).

### 3.3 LOB/LOD

The mean value of the blank was 2.72 pg/mL with a standard deviation of 1.96 pg/mL, and LOB was calculated as 5.95 pg/mL, which was rounded to 6 pg/mL. The mean and standard deviation of the spiked samples (C5–C6) were 14.4 and 3.60 pg/mL, respectively. The LOD for presepsin was calculated as 9.51 pg/mL, which was rounded to 10 pg/mL.

### 3.4 Method comparison

**FIGURE 2:**
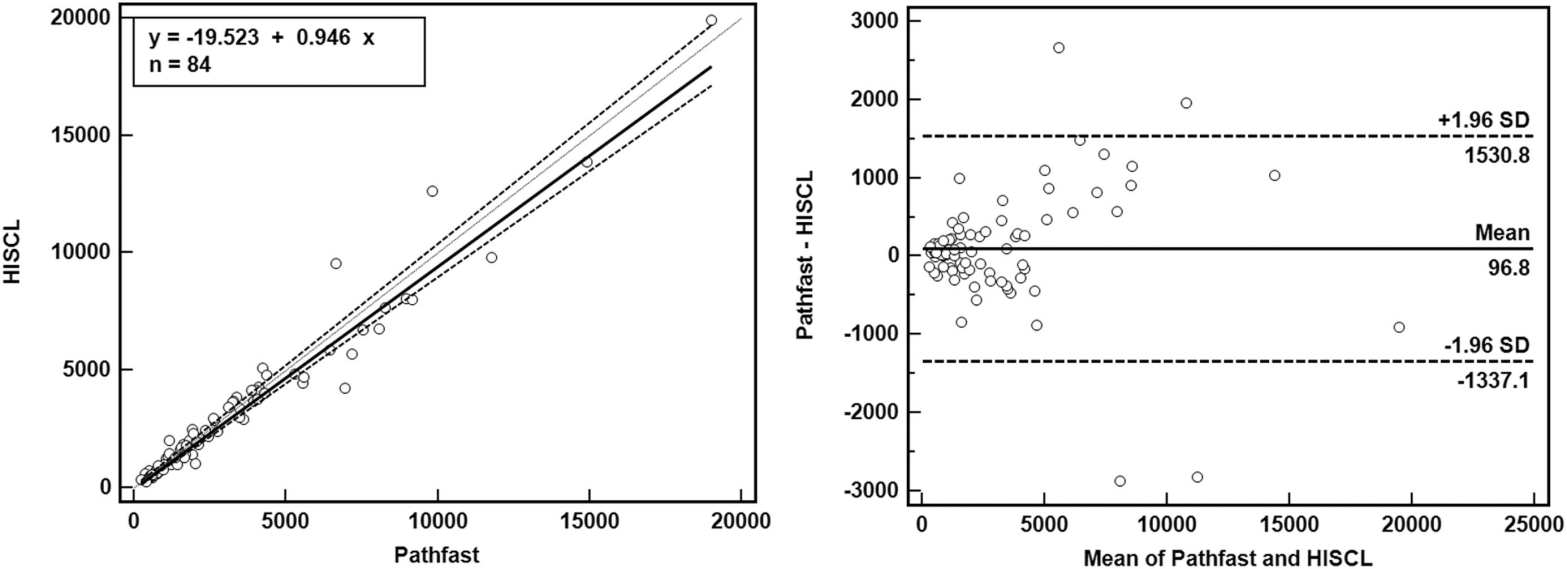
Method comparison of presepsin by HISCL‐5000 and PATHFAST using Passing–Bablock regression along with a Bland–Altman plot

Samples of 84 cases were measured for method comparison. Regression by Passing–Bablock analysis revealed the following: y = −19.522 + 0.945x (Syx = 534.7, R^2^ = 0.953). The correlation coefficient with a 95% confidence interval (95% CI) was 0.979 (0.967–0.986), with statistical significance (*p* < 0.001), a y intercept (95% CI) of −19.52 (−90.15–54.99), and a slope (95% CI) of 0.945 (0.905–1.000) (Figure 2). Bland–Altman plot revealed the differences between the methods against averages of values by two methods. Except for two data points, most of the data were within limits of agreement, which was defined as mean difference (± 1.96 * standard deviation).

### 3.5 Reference interval

A total of 120 healthy normal subjects were analyzed to determine the reference interval (Table 2). The upper limit of the reference interval was 299.75 pg/mL with a 95% confidence interval of (271–330 pg/mL); therefore, 300 pg/mL was used as a cut off value for positive results, which was in line with the manufacturer's claim.

**TABLE 2:** Laboratory data for normal healthy subjects for reference intervals and Pearson's correlation coefficients (*r*_xy_) with presepsin (*n* = 120)

	Mean	SD	min	max	*r*_xy_	*p value*
Presepsin (pg/mL)	151.6	66.7	68	359		
White blood cell (x10^9^/L)	5.79	1.51	3.41	10.51		
Red blood cell (x10^12^/L)	4.71	0.49	3.5	5.87		
Hemoglobin (g/dL)	14.1	1.61	9.3	18		
Hematocrit (%)	42.7	4.14	30.6	52.5	0.198	0.03
Platelet (x10^9^/L)	253	56.5	128	496		
Segmented neutrophil (%)	55.1	9.6	35.7	78.9		
Lymphocyte (%)	35.2	8.5	15.3	55.4		
Monocyte (%)	7.1	3.3	3.3	32.7		
Eosinophil (%)	1.9	1.4	0	6.7		
Basophil (%)	0.6	0.3	0	1.7		
Absolute neutrophil count (x10^9^/L)	3.2	1.3	1.2	8.3		
MCV (fL)	90.7	4.4	66.6	100.8		
MCH (pg)	30.1	1.9	18.8	34.5		
MCHC (%)	33.1	1.1	28.2	35.6		
Glucose (mg/dL)	101.3	23.3	71	245		
Creatinine (mg/dL)	0.83	0.24	0.46	1.95		
eGFR(CKD‐EPI) (mL/min/1.73m^2^)	93.6	18.9	32.9	133.6	−0.240	0.0129
IDMS traceable MDRD‐eGFR	88.9	21.7	17.8	144	−0.194	0.048
(mL/min/1.73 m2)						
AST (U/L)	23.6	8.3	10	55		
ALT (U/L)	23.4	13.6	6	83		
GGT (U/L)	35.5	40.3	8	217		
Total cholesterol (mg/dL)	200.3	52.7	62	517		
Triglycerides (mg/dL)	118	132	21	647		
HDL‐cholesterol (mg/dL)	58	12	32	96		

Abbreviation: ALT, alanine aminotransferase; AST, aspartate aminotransferase; CKD‐EPI, chronic kidney disease epidemiology collaboration; eGFR, estimated glomerular filtration rate; GGT, gamma glutamyl transferase; HDL, high density lipoprotein; IDMS, isotope dilution mass spectrometry; max, maximum; MCH, mean cell hemoglobin; MCV, mean corpuscular volume; MDRD, modification of diet in renal disease; min, minimum SD, standard deviation.

**FIGURE 3:**
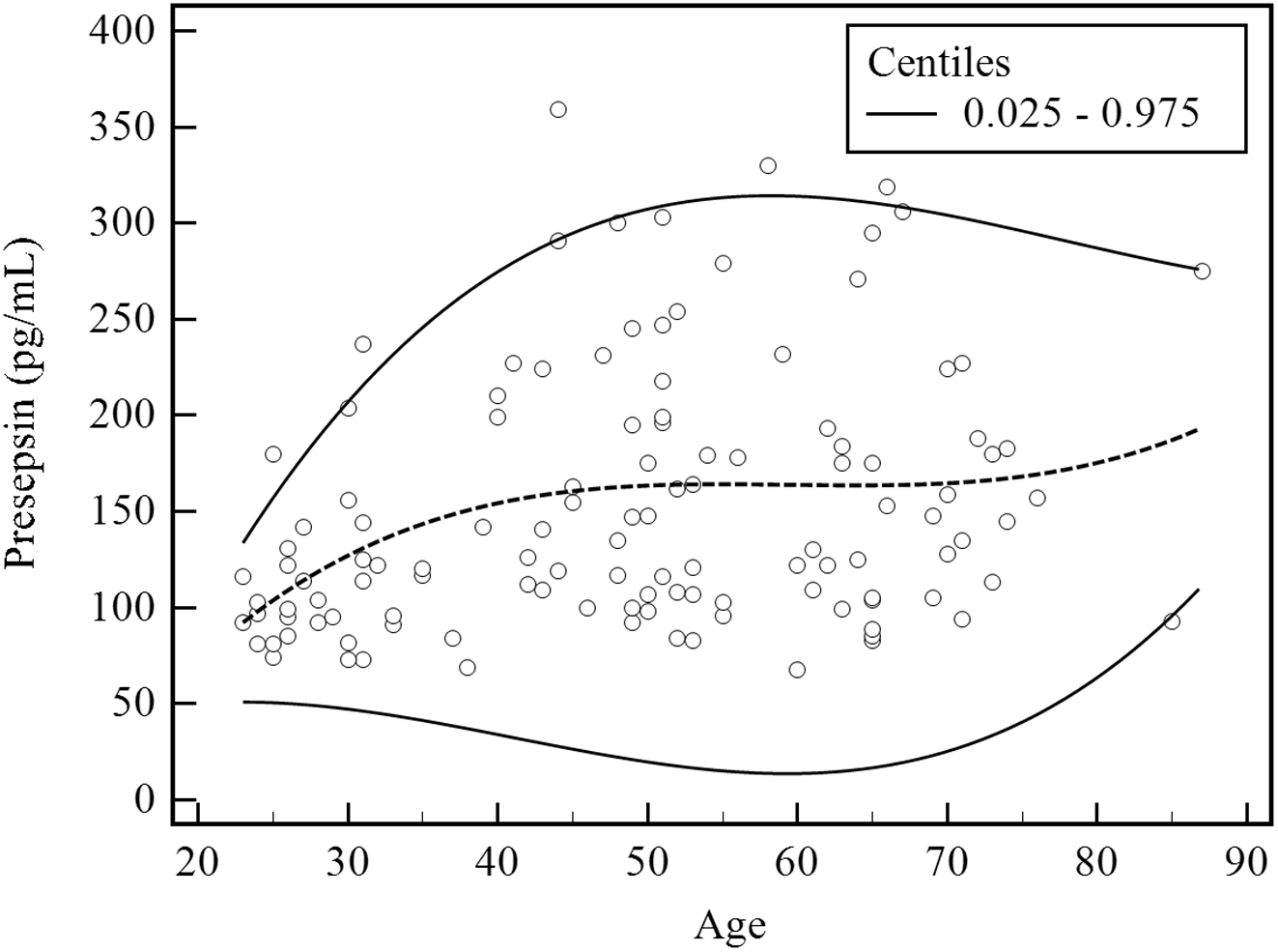
Relationship between age and presepsin. The dotted and continuous lines represent median values and 0.025 and 0.975 centiles, respectively (*n* = 120)

A regression model between age and reference interval was presented in Figure 3. The 0.025 and 0.975 centiles with estimated presepsin were measured for the following ages: 31–40, 47.2–207.3 pg/mL; 41–50, 33.9–274.9 pg/mL; 51–60, 19.7–307.4 pg/mL; 61–70, 13.7–314.1 pg/mL; 71–80, 25.3–304.1 pg/mL; 81–90, 63.9–286.9 pg/mL, respectively. For females (*n* = 64), the median and range of presepsin were 119.5 (69–330) pg/mL, and those for males (*n* = 56) were 150.5 (68–359) pg/mL, respectively (Figure S1).

**FIGURE 4:**
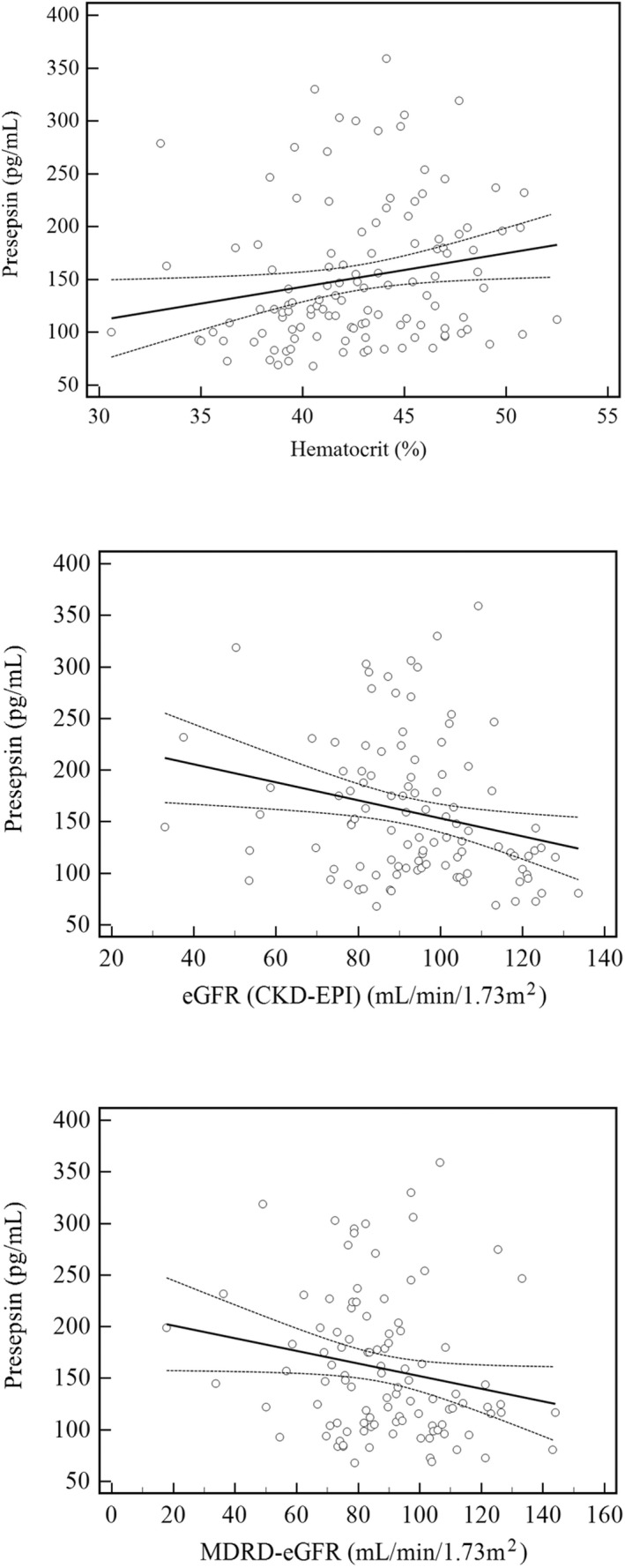
Correlation analysis between presepsin and hematocrit, eGFR (CKD‐EPI), and MDRD‐eGFR. Regression fit was as follows: hematocrit, y = 3.175x + 16.1 (r = 0.200, *p* = 0.030); eGFR (CDK‐EPI), y = −0.872x + 240.5 (r = 0.240, *p* = 0.0129); and MDRD‐eGFR, y = −0.612x + 213.3 (r = 0.190, *p* = 0.048). The continuous line represents regression fit, with the dotted line denoting a 95% confidence interval

Presepsin showed a positive correlation with hematocrit, with a correlation coefficient (*p* value) of 0.198 (*p* = 0.0301). The log value of presepsin showed a negative correlation with eGFR (CKD‐EPI) with a correlation coefficient (*p* value) of −0.270 (*p* = 0.005). Presepsin also showed a negative correlation with MDRD‐eGFR with a correlation coefficient (*p* value) of −0.194 (*p* = 0.048) (Figure 4).

### 3.6 Clinical presepsin data

Presepsin results measured from 2020 to 2021 was retrieved from the hospital electronic medical records. As expected, the department of internal medicine, infection division (MI) showed the highest mean values followed by the nephrology division (MN). These results indirectly reflected that the renal function influenced presepsin concentrations (Table 3, Figure S2).

**TABLE 3:** Normal or abnormal values of presepsin by the department (*n* = 1090). Age and presepsin concentrations are described as mean and standard deviation

Dept	*N*	Female (*N*)	Male (*N*)	Age (year)	Presepsin (pg/mL)
CS	24	1	23	63.0 ± 8.5	2230.8 ± 4241.0
ED	73	37	36	40.4 ± 30.4	731.3 ± 902.2
GS	627	258	369	64.3 ± 15.3	1132.6 ± 1714.3
MC	14	3	11	64.4 ± 10.2	659.9 ± 1261.5
MG	32	14	18	63.0 ± 16.6	3347.8 ± 5277.7
MH	41	22	19	53.8 ± 15.8	3393.2 ± 3558.3
MI	24	12	12	63.0 ± 10.6	4310.2 ± 6139.5
MN	25	11	14	59.8 ± 19.1	4043.0 ± 5089.1
MO	22	7	15	68.0 ± 9.8	3247.8 ± 4380.8
MP	208	75	133	68.6 ± 14.7	2305.4 ± 3708.6
	1090	440	650	62.9 ± 17.7	1676.0 ± 2929.1

Abbreviation: Dept, department; CS, chest surgery; ED, emergency department; GS, general surgery; MC, cardiology; MG, gastroenterology; MH, hematology; MI, infection; MN, nephrology; MO, oncology; MP, pulmonology.

## 4 DISCUSSION

Diagnosis of sepsis by SEPSIS‐3 was based on the SOFA score equal to or greater than 2 from the baseline along with the microbiological infection evidence. As scoring system is relatively complex, surrogate biomarkers have been studied to diagnose sepsis more feasibly within a limited time. Therefore, further studies are required for the prognosis or diagnosis of sepsis using biomarkers including presepsin.^1^, ^2^

Quick diagnosis should be made for the sepsis and a point of care test (POCT), which was performed at the bedside, might be more suitable for sepsis diagnosis. However, presepsin is a relatively new marker that requires a centralized analyzer along with the POCT. Generally, a centralized analyzer was regarded as more robust and stable than the POCT. A centralized analyzer could be regarded as a standard method that could support the POCT analyzer and in the case of presepsin, a POCT analyzer should also be developed.

The analytical performance of presepsin along with a method comparison was performed for the first time here. Presepsin is a relatively newly developed biomarker for sepsis compared to other biomarkers such as PCT, CRP, cytokine, chemokine, and growth factors.^25^, ^26^ Therefore, the development of an automated immune analyzer and evaluation of analytical performance is rare.

A method comparison between Sysmex HISCL‐5000 and PATHFAST was performed for the first time here. Around 80 samples were used, and the correlation coefficient was 0.979, which showed a high correlation coefficient. The reference range of presepsin was 300 pg/mL or below. These results were in line with previous results in which the values ranged from 21.8 to 294.2 pg/mL.^7^, ^8^ However, there was a slight difference between females and males, with males showing higher values. In addition, patients in their 50s and 60s showed higher presepsin compared to other age groups.

The previous reports showed that the reference range showed no difference in sex and age.^27^ The discrepant result between this study and the previous study could not be explained, but this might be related to comorbidities activating innate immunity or decreased renal function. In addition, it is speculated that presepsin was vulnerable to vibration, and handling samples might have affected concentration and caused this difference. Further studies are required for presepsin and preanalytical errors. For older age groups, the presepsin level was decreased, which might be explained by decreased innate immune function. Previous reports showed that presepsin level was not associated with insulin resistance in populations, whereas white blood cell count, CRP, and IL6 were related to insulin resistance.^28^ Further studies are required to determine the significance of higher presepsin levels in healthy old age groups.

In addition, a previous report showed that presepsin concentration was correlated inversely with renal function among subjects with chronic kidney disease. In that study, healthy normal subjects revealed that presepsin was correlated inversely with renal function calculated by eGFR (CKD‐EPI) and MDRD‐eGFR.^29^ Unlike previous reports, hematocrit showed a positive correlation with presepsin, compared to an inverse correlation of hemoglobin in previous reports.^30^ As presepsin was discreted through the kidney, lower renal function increased presepsin concentration. This was also a problem with troponins and careful interpretations are being made for cardiac disease.^31^ Presepsin requires interpretation criteria for chronic renal disease patients, subjects with lower renal function and patients with renal replacement therapy.

Patients with acute kidney injury might show elevated presepsin irrespective of microbial infection. As urinary tract infection is common in sepsis patients,^3^, ^4^, ^5^, ^6^ presepsin concentrations with underlying renal disease should be carefully interpreted because the bacterial infection could be masked by elevated presepsin. Patients with anemia or with low hemoglobin and hematocrit levels should be studied for presepsin for accurate interpretation.

The limitation of this study is that there were no desirable specifications of imprecision derived from biological variation. Trueness verification was unable to be performed due to a lack of reference standard materials. As few patient samples were available, a calibrator was used for linearity evaluation, which might have eliminated matrix effects.

In conclusion, presepsin analyzed by HISCL‐5000 showed reliable analytical performance that could be used in clinical settings. A wide range of analytical measurements ranging from 200 to 30,000 pg/mL was verified. As presepsin showed positive and negative correlations with hematocrit and renal function, careful interpretation is required for patients with underlying diseases involving red blood cells or kidneys. Further studies are required for trueness verification and possible age differences in healthy normal control groups.

## CONFLICT OF INTEREST

The authors state no conflict of interest.

## PATIENT CONSENT STATEMENT

Informed consent was waivered by the institutional review of the board due to the usage of anonymous leftover samples.

## Supporting information


Appendix S1

## Data Availability

All the data are presented in the manuscript and in supplementary materials.
