# Supplementary material for: Performance evaluation of presepsin using a Sysmex HISCL‐5000 analyzer and determination of reference interval
Source: J Clin Lab Anal. 2022 Jul 23;36(9):e24618. doi: 10.1002/jcla.24618 (PMC9459287; doi:10.1002/jcla.24618)
Supplement: Supplementary file 1 — Appendix S1 [file JCLA-36-e24618-s001.docx]

Supplementary Figure1. Reference ranges of females and males. The median value of

presepsin with the lowest and highest values in femaleswas 119.5 (69- 330) and in maleswas 150.0 (69-359).


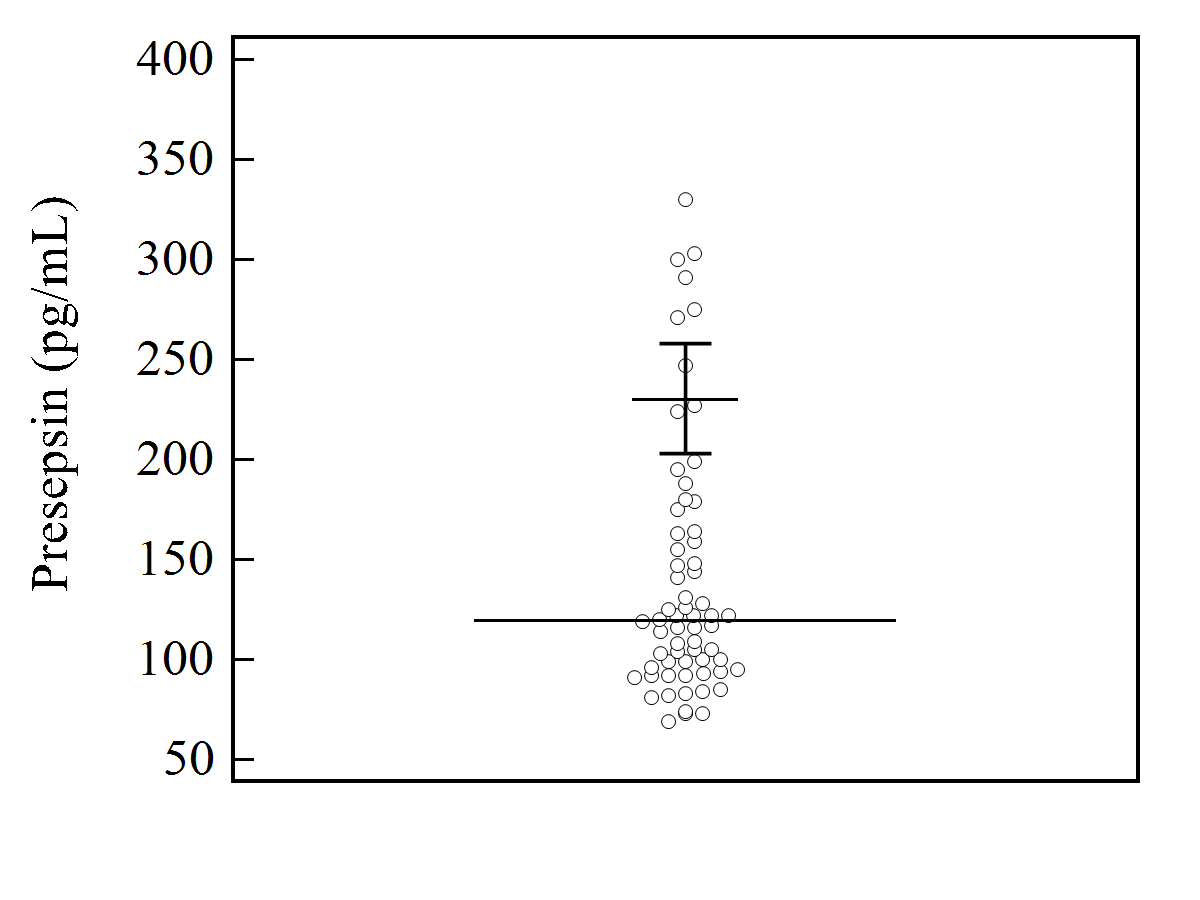

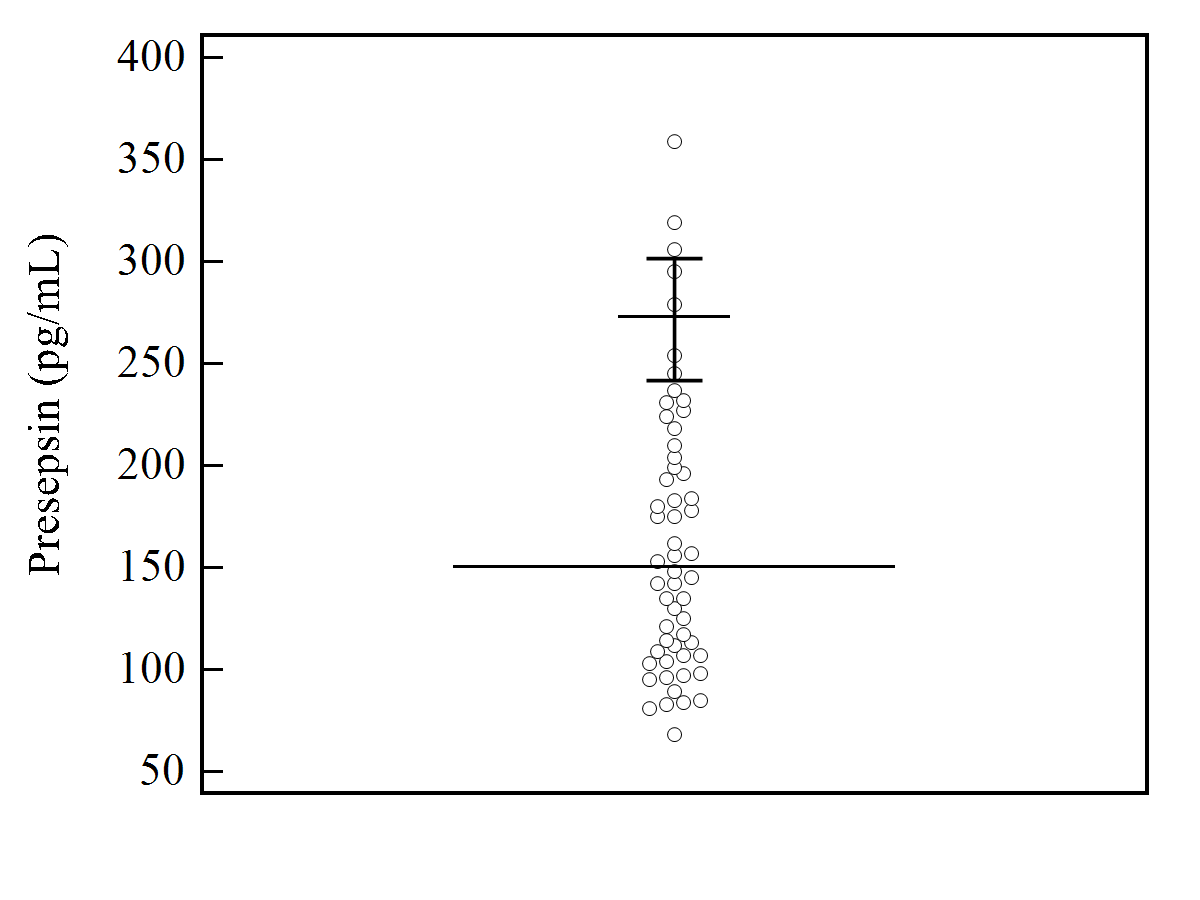


Female

Male

Supplementary Figure 2. Flow chart for patients with presepsin by department

Abbreviation: CS, chest surgery; ED, emergency department; GS, general surgery; MC, cardiology; MG, gastroenterology; MH, hematology; MI, infection; MN, nephrology; MO, oncology; MP, pulmonology; Dept, department; ME, endocrinology; MR, rheumatology; MU, general; NP, psychiatry; NS, neurosurgery; NU, neurology; OBGY, obstetrics and gynecology; OS, orthopedic surgery; UR, urology.
